# 

**Published:** 1933

**Authors:** 


### CONTENTS.

*Page*

The Twenty-second Long Fox Memorial Lecture: Recent Advances in the Ætiology, Diagnosis and Treatment of Cancer. By Cecil A. Joll, M.D., M.S., F.R.C.S. .. .. .. .. 201

The Present Position of Radium Therapy. By Sylvia B. Wigoder, M.D., Ph.D., D.M.R.E. .. .. .. .. .. .. 233

Bacillus Coli Infections of the Female Urinary Tract. By H. J. Drew Smythe, M.D., M.S., F.R.C.S., F.C.O.G. .. .. .. .. 243

The Use of the Ventral Position in the Treatment of Tuberculous Disease of the Spine in Children. By K. H. Pridie, M.B., B.S., F.R.C.S. 261

Bristol University: Department of Preventive Medicine .. .. 267

Reviews of Books .. .. .. .. .. .. .. .. 271

Obituaries. William Alexander Smith, M.A., M.B., M.R.C.S., F.C.S. 286
Charles Albert Calmette .. .. .. .. .. 287

Meetings of Societies .. .. .. .. .. .. .. 293

The Medical Library of the University of Bristol .. .. .. 297

Publications Received .. .. .. .. .. .. .. 300

Local Medical Notes .. .. .. .. .. .. .. 302

